# Cognitive and neural underpinnings of friend-prioritization in a perceptual matching task

**DOI:** 10.1093/scan/nsaf009

**Published:** 2025-01-20

**Authors:** Tianyu Gao, Yuqing Zhou, Xinyue Pan, Wenxin Li, Shihui Han

**Affiliations:** Department of Psychology, Faculty of Arts and Sciences, Beijing Normal University at Zhuhai, 18 Jinfeng Road, Zhuhai 519087, China; Beijing Key Laboratory of Applied Experimental Psychology, National Demonstration Center for Experimental Psychology Education, Faculty of Psychology, Beijing Normal University, No. 19 Xinjiekouwai Street, Beijing 100875, China; CAS Key Laboratory of Behavioral Science, Institute of Psychology, Chinese Academy of Sciences, 16 Lincui Road, Beijing 100101, China; School of Management, Economics and Shenzhen Finance Institute, The Chinese University of Hong Kong, 2001 Longxiang Boulevard, Longgang District, Shenzhen 518172, China; School of Psychological and Cognitive Sciences, PKU-IDG/McGovern Institute for Brain Research, Peking University, 52 Haidian Road, Beijing 100080, China; School of Psychological and Cognitive Sciences, PKU-IDG/McGovern Institute for Brain Research, Peking University, 52 Haidian Road, Beijing 100080, China

**Keywords:** friend-prioritization, drift diffusion model, EEG, shape-label matching task, self-prioritization

## Abstract

Previous findings of better behavioral responses to self- over other-related stimuli suggest prioritized cognitive processes of self-related information. However, it is unclear whether the processing of information related to important others (e.g.friends) may be prioritized over that related to the self in certain subpopulations and, if yes, whether friend-prioritization and self-prioritization engage distinct cognitive and neural mechanisms. We collected behavioral and electroencephalography (EEG) data from a large sample (*N* = 1006) during learning associations between shapes and person labels (self or a friend). Analyses of response times and sensitivities revealed two subpopulations who performed better to friend–shape or self–shape associations, respectively (*N* = 216 for each group). Drift diffusion model (DDM) analyses unraveled faster information acquisition for friend–shape (vs. self–shape) associations in the friend-prioritization group but an opposite pattern in the self-prioritization group. Trial-by-trial regression analyses of EEG data showed that the greater amplitudes of a frontal/central activity at 180–240 ms poststimulus were correlated with faster information acquisition from friend–shape associations in the friend-prioritization group but from self-shape associations in the self-prioritization group. However, the frontal/central neural oscillations at 8–18 Hz during perceptual learning were specifically associated with speed of information acquisition from friend–shape associations in the friend–prioritization-group. Our findings provide evidence for friend-prioritization in perceptual learning in a subpopulation of adults and clarify the underlying cognitive and neural mechanisms.

## Introduction

A wealth of empirical research has shown evidence that people prioritize information related to the self over that pertaining to others (i.e. “the self-priority effect”). For instance, adults remember better (Symons and Johnson 1997, Conway and Pleydell-Pearce 2000, Cunningham et al. 2008, Yin et al. 2019) and pay more attention to self-related (vs. other-related) information (Turk et al. 2011). They respond faster and more accurately to images of their own compared to others (even close others such as friends) faces/names (Brédart et al. 2006, Ma and Han 2010, Alexopoulos et al. 2012). The self-priority effect is also evident in a perceptual matching task that requires responses to learned associations between shapes (e.g. triangle, square, or circle) and personal labels (e.g. self, friend, or stranger) (Sui et al. 2012). Using this shape-label matching task, researchers have repeatedly reported shorter reaction times (RTs) and higher response accuracies in response to correct self–shape associations than friend or stranger associations (Sui et al. 2012, Siebold et al. 2015, Schäfer et al. 2016, 2020, Reuther and Chakravarthi 2017, Desebrock et al. 2018, Macrae et al. 2018, Woźniak et al. 2018, Woźniak and Knoblich 2019, Hu et al. 2020). While the findings demonstrate a key role of self-relevance in modulations of cognitive and neural processes of information in various contexts (Northoff et al. 2006, Northoff 2011, Qin and Northoff 2011), it remains unclear whether information related to important others such as friends may be prioritized over that about the self in subpopulations and, if yes, whether friend-prioritization and self-prioritization engage distinct cognitive and neural underpinnings. The processes of the self and others constitute the two core components of social cognition (Lieberman 2007). Despite the previous findings of self-advantage in social information processing, it is still unresolved whether self-prioritization characterizes human social cognition universally. This can be clarified by testing the existence of friend-prioritization in subpopulations. In addition, to clarify both common or distinct neurocognitive mechanisms underlying self-prioritization and friend-prioritization at the group level would offer new insights into group-level differences in social cognitive and affective processes that are crucial for social relationships and behaviors.

Previous findings of several lines of research have provided a hint for the advantage of others over the self in human behavior and cognition in a small sample. First, altruistic behavior that benefit others at the cost of self-interest has been observed in human societies (Batson and Powell 2003). There are even extraordinary altruists, though a limited number of cases, who placed others’ interests absolutely over their own (e.g. volunteered to donate a kidney to a stranger) and showed specific patterns of brain activities (e.g. enhanced amygdala volume and responses to other’s fearful facial expressions) (Marsh et al. 2014). Second, there has been behavioral evidence for the advantage of perceptual processing of significant others’ (supervisors’) faces over one’s own faces (e.g. Ma and Han 2009). Brain imaging research also found overlapping neural representations of the self and close others in specific brain regions in a specific cultural group (e.g. the medial prefrontal cortex in a self-reference task, Zhu et al. 2007, Han et al. 2016). Third, recent work using the shape-label matching task found that the prioritization effect on behavioral performances may emerge for either self-related or friend-related stimuli that are encountered most frequently (Falbén et al. 2020, Svensson et al. 2023), indicating flexible priority of processing goal-relevant stimuli irrespective of whom the stimuli are associated with. Together, these findings suggest that, if prioritization of self-related or other-related information processing depends upon social contexts and experiences, there might be subpopulations that prioritize the processing of information related to significant others (e.g. friends) over self-related information due to specific social experiences. This hypothesis is consistent with the self-determination theory (Deci and Ryan 2012) which claims that humans are motivated to act in ways to satisfy the basic psychological needs of autonomy, competence, and relatedness. Close and supportive relationships contribute to our psychological well-being and a sense of belonging. Friend-prioritization may satisfy the need for relatedness and nurture social bonds that are critical to mental health of a subpopulation of humans. Indeed, a recent study of real-world altruists reported reduced personal distress in prosocial individuals who exhibited high valuation of others’ outcomes in social decision making (Rhoads et al. 2023).

Previous studies of self-prioritization usually tested a small sample and examined averaged behavioral responses to self-related/friend-related stimuli across individuals. In this approach, individuals who exhibited friend-prioritization in behavioral responses were usually concealed by the analyses of the mean values of different individuals’ performances or treated as outliers. A challenge to verify friend-prioritization at the group level and to clarify relevant neural underpinnings is to collect behavioral and brain imaging data from a large sample to identify subpopulations that show friend-prioritization and self-prioritization, respectively. The current study collected behavioral responses and electroencephalography (EEG) signals during the shape-label matching task (Sui et al. 2012) from a large sample (*N* = 1006). This shape-label matching task measures RTs and response accuracies to recently learned self–shape and friend–shape associations to control effects of differential familiarity of conventional self- and friend-related stimuli such as faces and names. The large sample allowed us to identify friend-prioritization in behavioral performances in a sample larger than those tested in previous studies for self-prioritization. We calculated a model-free index of response efficiencies related to correct self–shape and friend–shape associations (i.e. the response efficiency score = RTs/response accuracy, Townsend and Ashby 1978) and the differential response efficiency score between self–shape and friend–shape associations for each participant. A differential response efficiency score larger than zero defines the friend-prioritization group (FP-Group), whereas a differential response efficiency score smaller than zero defines the self-priority group (SP-Group). Similar to previous research (Sui et al. 2012, Golubickis et al. 2018), we conducted signal detection theory (Green 1966) and drift diffusion model (DDM, Ratcliff 1978) analyses of behavioral performances in the shape-label matching task to examine latent cognitive processes that distinguish between two samples who showed friend-prioritization or self-prioritization. Four latent cognitive processes can be captured by the parameters of DDM analyses. These include the drift rate (*v*, an index of speeds of evidence acquisition, a larger drift rate corresponds to a quicker information update), the boundary separation (*a*, an index of decision strategies, a larger distance between the two boundaries suggests a more conservative strategy of decision making), the starting point (*z*, an index of the relative amount of evidence needed for each response), and nondecision time (*t*_0_, an index of the duration of preparatory process and postdecisional phase) (Ratcliff and McKoon 2008). We also collected measures of interpersonal traits (e.g. self-construals, Singelis 1994) and empathic ability (Davis 1983) and participants’ relationships with friends to assess whether and how self-/friend-prioritization varied along interpersonal traits/relationships across different individuals.

Self-prioritization in the shape-label matching task has been associated with specific brain activities. A functional MRI study found increased activity in the ventromedial prefrontal cortex (VMPFC) in response to correct self-shape compared to stranger-shape associations (Sui et al. 2013). The VMPFC displayed enhanced functional connectivity with the frontoparietal cortex during maintenance of learned self-associated (vs. other-associated) cues (Yin et al. 2021). These findings support the general functional role of the VMPFC in self-related processing (Northoff et al. 2006, Northoff 2011, Qin and Northoff 2011). A recent work probed the temporal properties of neural processes involved in self-prioritization by recording EEG from twenty participants during the shape-label matching task (Sui et al. 2023). It was found that a negative activity peaking around 150 ms after stimulus onset over the lateral temporal electrodes (N1) was enlarged to learned self–shape compared to friend–shape (or stranger–shape) associations but did not differ between friend–shape and stranger–shape associations. A similar pattern was observed for the amplitude of a late positive activity at 340–500 ms over the parietal electrodes (P3). These EEG findings suggested that multiple-level neural processes may contribute to self-prioritization in perceptual matching performances. Based on the previous EEG findings, the current work analyzed EEG data to investigate neural correlates of friend-prioritization and self-prioritization shown in behavioral performances. We sought to clarify neural activities that are common or distinct for friend–shape and self–shape associations by comparing the results of behavioral and EEG data analyses between FP-Group and SP-Group in the shape-label matching task. Our findings revealed both common and distinct cognitive and neural substrates of friend-prioritization and self-prioritization in the perceptual matching task.

## Methods

### Participants

Behavioral and EEG data were collected from 1006 Chinese university students (149 males, 850 females, age: mean ± s.d = 20.5 ± 1.12, 7 participants did not provide demographic information). Seventeen participants were excluded from data analyses due to their failures of the experiment procedures. This sample size was not predetermined. We recruited as many participants as possible in 2 academic years. All participants self-reported no neurological diagnoses, had normal or corrected-to-normal vision, and were paid for their participation. Written informed consent was obtained prior to the experiment. This study was approved by the local ethics committee at the School of Psychological and Cognitive Sciences, Peking University.

We calculated response efficiency (i.e. RTs/accuracy), which is a model-free behavioral index that considers the speed-accuracy trade-offs in the shape-label matching task (Sui et al. 2013), to classify participants characterized by friend-priority (behavioral efficiency related to friend–shape pairs outperformed that related to self–shape pairs in the matching condition), or self-priority (behavioral efficiency related to self–shape pairs outperformed that related to friend–shape pairs in the matching condition). The difference in response efficiency (self–shape pairs minus friend–shape pairs) was used to define the friend-priority and self-priority groups. A differential response efficiency score larger than zero was used as the selection criteria for the friend-priority group (FP-Group), resulting in 216 participants for the FP-Group. For statistical comparisons we selected the same number of participants with the most prominent self-prioritization effect in their behavioral performances as a control group (the self-priority group or SP-Group). The participants with the most prominent self-prioritization effect were selected for SP-Group so that the comparison of behavioral and EEG results between FP-Group and SP-Group would allow us to find neurocognitive mechanisms that contrast FP-Group and SP-Group to a maximum degree. Behavioral and EEG results were compared between FP-Group and SP-Group to elucidate their distinct cognitive and neural mechanisms. See Supplementary Table S1 for the exact number of participants of each group after exclusion due to missing EEG data, questionnaires, or economics game data.

After data collection, we conducted a power analysis to validate the adequacy of current sample sizes. Given the theoretically smallest but informative effect size (*f* = 0.1), a sample size of 266 participants was required to obtain a small effect size of 0.1, with type I error of 5% and power of 90% for a within–between interaction of a repeated-measures analysis of variance (ANOVA). The result suggests that the sample sizes of 432 in the 2 subject groups provides sufficient power for testing our hypotheses.

### Stimuli and procedure

Before EEG recording, participants reported their demographic information (e.g. gender, age, major, and ethnic) and completed the Self-Construal Scale (Singelis 1994) and the Interpersonal Reactivity Index (IRI, Davis 1983) to assess their self-construals regarding independence/interdependence and their social sensitivity to others’ emotional states (i.e. empathic concerns, perspective taking, fantasy, personal distress). Each participant was asked to give a same-gender friend’s name and to evaluate their friendships with friends by reporting how long they had known each other and their closeness, likability, and familiarity on a Likert scale (1 = not at all, 7 = extremely). Each participant also selected a stranger’s name from a list of 20 same-gender names that would be used in the stranger condition in the following task.

After EEG recording, participants were asked to finish two one-shot economic games, i.e. the trust game (Berg et al. 1995) and public goods game (Ledyard 1995), to assess their social trust and cooperative tendencies (Andreoni et al. 2010). Participants were told they were paired with mutually anonymous partners in each game. In the trust game, “the sender” (i.e. the participant) initially received 10 monetary units and had to decide how much to give to the partner (i.e. “the receiver”) who would be able to invest and triple the received money. The receiver then decided how much money to send back to the sender. The final payoff for the sender was the sum of the money left for oneself and the money returned from the receiver. Social trust was quantified by a sender’s investment. In the four-player public goods game, each participant initially received 80 monetary units and had to decide how much to contribute to a project conducted by four players. The money contributed to the common project would be doubled and evenly divided among the four players. The final payoff for each participant was the sum of the money left for oneself and the money returned from the common project. Cooperative tendencies were quantified by a participant’s contributions to the common project.

### Shape-label matching task

**Figure 1.**
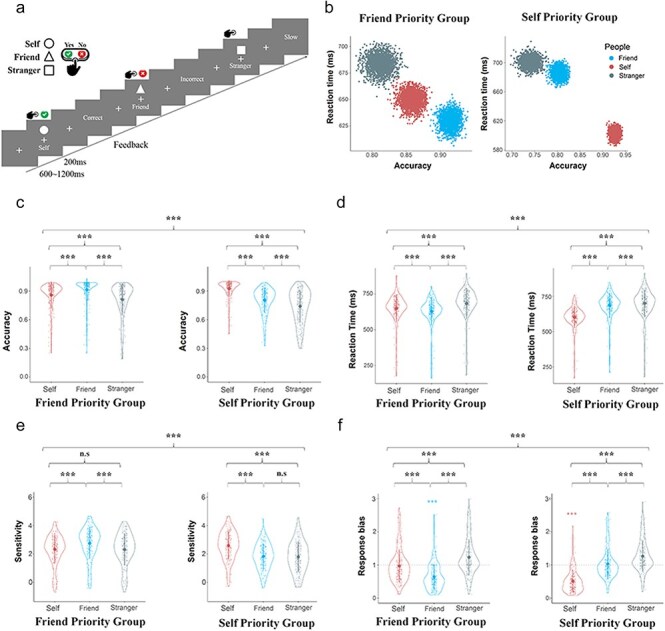
Experimental procedures and behavioral results. (a) Illustration of the experimental procedures of the associative learning task. Shapes assigned to different labels (Self, Friend, and Stranger) were counterbalanced across participants. (b) Distributions of bootstrapped sample means for matching trials in FP-Group (left panel) and SP-Group (right panel). (c) and (d) Combined response accuracies and reaction times of self, friend , and stranger match associations in FP-Group (left panel) and SPGroup (right panel) during the associative learning task. Parameters of signal detect theory. (e) and (f) The results of sensitivity (d’) and response biases (β) of the signal detection theory analysis. Shown are group means (big dots), standard deviations (bars), measures for each individual (small dots), and distribution (violin shape).

Behavioral and EEG indices of self-prioritization were assessed using the shape-label matching task (Sui et al. 2012). Before this task, each participant was asked to report a name of a same-gender friend and selected a stranger’s name from a list of 20 same-gender names. During the label–label matching task, participants were informed that they had to remember the assigned associations between three words (i.e. “self,” “friend,” and “stranger”) and three shapes (i.e. triangle, square, and circle) within 1 min “self,” “friend,” and “stranger” indicated oneself, the friend, and the stranger, respectively. The associations between words and shapes were counterbalanced across the participants. Thereafter, the participants were presented with word–shape pairs and had to perform judgements on whether a word–shape pair represented a correct association while EEG signals were recorded. As shown in Fig. 1a, on each trial, a word–shape pair was displayed for 100 ms, which was followed by a fixation cross with a duration varying randomly from 900 to 1700 ms. A feedback word (“correct,” “incorrect,” or “slow”) was presented on the screen for 500 ms to indicate correct/incorrect responses or slow responses (i.e. without a response within 1100 ms after the presentation of a word-shape pair, similar to previous work (Sui et al. 2013). Participants were asked to respond as quickly and accurately as possible by pressing one of two buttons (left vs. right) using index and middle fingers of the right hand. The word–shape pairs were the same as the participants learned in half of the trials (i.e. the matching condition) but were not in other half of the trials (i.e. the nonmatching condition in which a word was presented with an incorrect shape). Stimuli in the match and nonmatching conditions were presented in a random order. The relationships between responding fingers (index or middle fingers) and buttons (“yes” or “no”) were counterbalanced across the participants. There were 110 trials in each of the six conditions (i.e. three words (self/friend/strange) were associated with correct/incorrect shapes).

### Bootstrapping procedure of reaction time and accuracy

We adopted a standard bootstrapping procedure of accuracy and reaction time to illustrate the distinct patterns of behavioral responses (accuracy and reaction time) between FP-Group and SP-Group. From the data set of accuracy and reaction time to self-matching, friend-matching, and stranger-matching word–shape pairs in SF-Group and FP-Group, a bootstrapped data set in each condition was nonparametrically resampled with replacement. The mean of this bootstrapped sample was then calculated and plotted as one of the points (*x*, *y*) in a panel with the horizontal (*x*) and vertical (*y*) axes showing accuracy and reaction time, respectively. The same procedure was repeated for *n* = 1000 times to estimate the population means and variations in each condition.

### Signal detection theory modeling of behavioral responses

We calculated sensitivity (d’) and response bias (β) based on the “signal detection theory” (Green 1966) separately for responses in each condition. d’ was estimated as the difference between *z*-scores (i.e. the inverse of the standard normal cumulative distribution) of hit and false alarm rates (Macmillan 1993). A larger d’ indicates a greater ability to distinguish match from nonmatching pairs.


$$d{^{^{\prime}}} = \,{{\Phi }^{ - 1}}\left( H \right) - {{\Phi }^{ - 1}}\left( F \right)$$

β was calculated by squaring the *z*-scores that corresponds to the false-alarm rate, subtracting the square of the *z* score that corresponds to the hit rate, and dividing the result by 2, then, power the result with exponential function (Stanislaw and Todorov 1999). A β larger than 1 indicates a greater tendency toward a “no” response, while a β smaller than 1 suggests a bias toward a “yes” response.


$$\beta ={{e}^{\genfrac{}{}{.3pt}{2}{{{{\left( {{{\Phi }^{ - 1}}\left( F \right)} \right)}^2} - {{\left( {{{\Phi }^{ - 1}}(H)} \right)}^2}}}{2}}}$$

### Hierarchical drift diffusion modeling of behavioral responses

We conducted DDM analyses (Ratcliff 1978) of behavioral performances (including both response accuracies and RTs) to examine potential latent cognitive processes that support selections of one of two simple options and are captured by several parameters (Ratcliff and McKoon 2008). The drift rate (*v*) in the DDM represents the speed of evidence acquisition (a larger drift rate corresponds to a quicker information update) and reflects a stimulus evaluation process determined by stimulus attributes (White and Poldrack 2014). Responses are initiated immediately by the accumulative evidences across one of two threshold boundaries. The distance between the two boundaries (*a*) in the DDM represents decision strategies (a larger threshold distance suggests a more conservative strategy of decision making). The starting point (*z*) in the DDM indicates the position where evidence accumulation begins and reflects the relative amount of evidence needed for each response (*z* larger than a/2 suggests that less (or more) evidences are required to reach the upper (or lower) boundary). The nondecision time (*t*_0_) in the DDM captures the duration of preparatory process (i.e. stimuli perceptual input) and postdecisional phase (i.e., movement initiation and execution). Behavioral responses were fitted into the model using the hierarchical Bayesian implementation of the hierarchical drift diffusion model (HDDM-0.6.0) toolbox (Wiecki et al. 2013) that assumes that the model parameters for individuals are randomly sampled from group distributions and both group- and individual-level parameters were estimated by Bayesian statistical methods (Vandekerckhove et al. 2011).

Similar to the previous work (Golubickis et al. 2017, Gao and Han 2023), the behavioral data were fitted to the DDM with a “Yes” response toward the upper threshold and a “No” response toward the lower threshold. Five percent of trials with the longest response were treated as outliers by estimating a mixture model that enables stable parameter estimation even with outliers present in the data. The precise value of the percentage of outlier does not affect the outcome and values >0.001 and >0.1 are sufficient to capture the outlier (https://hddm.readthedocs.io/en/latest/howto.html#outliers). We estimated drift rates (*v*) in different conditions, respectively (Labels: Self vs. Friend vs. Stranger × Matching Condition: Match vs. Nonmatch) for each group (SP-Group vs. FP-Group) to captures the bias induced by stimulus property (White and Poldrack 2014). The nondecisional time (*t*_0_) was estimated separately for the match and nonmatching conditions for each group (SP-Group vs. FP-Group) to account for response preparation and execution that were facilitated vs. delayed in the match compared to nonmatching conditions (Voss et al. 2013). To assess whether self- and friend-priority in behavioral responses were elicited by initial predilection, we estimated starting point (*z*) as function of different targets (Self, Friend, Stranger) in different groups (SP-Group vs. FP-Group). To examine whether distinct strategies (cautious vs. hurrying) were adopted to make final judgement between two groups, we compared the boundary separation (a) for responses to different targets (Self, Friend, Stranger) between the two groups (SP-Group vs. FP-Group). This hypothesized model was defined as Model 1. Bayesian posterior distribution of these parameters were modeled using the Markov Chain Monte Carlo (MCMC) algorithm. To improve the probability of convergence for samples in MCMC, 10 000 posterior samples were repeated and the first 1000 samples was discarded. To validate the adequacy and necessity of current model, we proposed additional 14 simpler candidate models [Model 7 is the same model identified in previous study (Golubickis et al. 2017)] and compared these models using deviance information criteria (DIC) (Spiegelhalter et al. 2002).To avoid redundancy of the hypothesized model (Model 1), we tested whether the simpler models performed better (with lower DIC values) than our hypothesized model in term of accounting for the observed data. These alternative models were more simpler than our hypothesized model by reducing the hypothesized effects on different parameters one-by-one. The results shown indicate that the DIC of our hypothesized model (Model 1) is less than all other candidate models (Model 2– 15, see Supplementary Table S2), suggesting that our hypothesized model best fitted the behavioral data than other models.

### EEG data acquisition and analysis

EEG was recorded from 30 Ag/AgCI scalp electrodes mounted in an elastic cap. The electrodes were arranged according to the international 10/20 system and online referenced to the Cz electrode. Eye blinks and vertical eye movements were recorded with two additional electrodes located above and below the left eye. The horizontal electro-oculogram was recorded from two additional electrodes placed on the left and right canthi. Electrode impedance was kept <5 kΩ. EEG was recorded continuously using a NeuroScan system with a band-pass filter (0.01–400 Hz) and a sampling rate of 1000 Hz. During offline processing EEG was rereferenced to the average of the left and right mastoid electrodes and band-pass filtered from 0.1 to 40 Hz. Epochs were extracted for word–shape pairs with each epoch starting from 200 ms prior to word–shape pair onsets and lasting for 1000 ms. Baseline corrections were conducted for neural activities in different conditions using the mean amplitudes of EEG signals in a 200 ms time window before stimuli onset. These epochs were subject to independent component analysis (ICA) using the EEGLAB toolbox (Delorme and Makeig 2004) to detect and remove artifacts including eye movements. Trials contaminated by residual eye movements and muscle artifacts exceeding ±70 μV at any electrode were removed before further analyses. These resulted in 92.45 ± 23.16 (mean ± s.d.) artifact-free trials per condition.

Event-related potentials (ERPs) to word–shape pairs were characterized by an early negative component at 100–150 ms (N1) and a positive component at 180–240 ms (P2) which peaks at frontal–central regions, a negative component at 240–300 ms (N2) peaks at central–parietal regions, a positive component at 320–360 ms (P3) over the parietal region, and a positive component at 500–700 ms (LPP) over central to parietal regions. ERPs to self–/friend–shape pairs in the matching condition were calculated, respectively. The time windows and clusters of channels of each component were defined based on voltage topographies of each component. The mean amplitude of the frontal N1 component, which has been associated with voluntarily selective attention to stimuli for further processing (Näätänen 1982), was calculated at a cluster of frontocentral midline electrodes (FCz, Fz, Cz) at 100–150 ms after stimuli onsets. The mean amplitude of the LPP component that is linked to neural responses to stimulus significance (Hajcak and Foti 2020) was calculated at a cluster electrodes covering the frontal, central to parietal regions (FZ, FC3, FC4, FCZ, C3, C4, CZ, CPZ) at 500–700 ms after stimuli onsets. As shown in Fig. 3, the P2, N2, and P3 amplitudes are greater for friend–pairs compared with self–pairs in both FP- and SP-Groups, indicating a main effect of other-processing (vs. self-processing). The mean P2 amplitude was calculated at a cluster of central to parietal electrodes (FC3, FC4, FCZ, C3, C4, CZ, CP3, CP4, CPZ) at 180–240 ms after stimuli onsets; the mean N2 amplitude was calculated at a cluster of the right central to temporal electrodes (P4, Pz, T4, T6, TP8, C4, Cz, CP4, CPZ) at 240–300 ms after stimuli onsets; the mean P3 amplitude was calculated at a cluster of the left parietal, temporal and occipital electrodes (P3, PZ, TP7, T5, CP3, O1, O2, OZ) at 320–360 ms after stimuli onsets.

Time-frequency analyses of nonphased locked activities were conducted based on a wavelet decomposition of the neural signal between 1 and 40 Hz in 1 Hz steps. Similar with previous studies (Mu et al. 2008, Gao et al. 2020), ERPs in each condition were subtracted from each corresponding EEG epoch before time-frequency analyses to remove the phase-locked EEG activity. The time series in each epoch was convolved with a set of complex Morlet wavelets *w* (*t*, *f*_0_), referred to as a Gaussian-shaped complex sine wave: exp(−*t*^2^/2σ*t*^2^)exp(2iπ*f*_0_*t*), where *t* is time, *f*_0_ is frequency, and σ is the cycle of each frequency band (Kronland-Martinet et al. 1987). Similar to previous studies (Mu et al. 2008, Mu and Han 2010), the wavelet family was defined by *f*_0_/σ*f* = 5, with f_0_ ranging from 1 to 40 Hz in 1 Hz steps.


$$w\left( {t,\,{f_0}} \right) = Aexp\left( {\frac{{ - {t^2}}}{{2{{\sigma }_t}^2}}} \right){exp}\left( {2i\pi {f_0}t} \right)$$

A is a normalization factor that equal to (${{\sigma }_t}\sqrt \pi $)^−1/2^,which makes total wavelet energy equal to 1. The convolution of complex wavelet with the signal provides a TF representation of signal, that can be used to estimate the instantaneous power *P*(*t*):


$$P\left( t \right) = real{\left[ {TF\left( t \right)} \right]^2} + imag{\left[ {TF\left( t \right)} \right]^2}$$

Next, the index of intra-epoch power change (event-related synchronization and desynchronization, ERS/ERD) was normalized by calculating the increased/decreased percentage, compared to the baseline power *P*_0_ (−300 to −100 ms), and *P*(*t*) refers to the spectrum power at time window *t* after stimulus onset.


$$ERS\,/ERD = \frac{{P\left( t \right) - {P_0}}}{{{P_0}}}$$

Using the fieldtrip toolbox (Oostenveld et al. 2011), we conducted whole-brain cluster-based permutation paired *t*-tests between nonphase-locked activities at 0–800 ms in response to correct self–shape and friend–shape associations, including delta/theta (2–7 Hz), alpha and low beta (8–18 Hz), and beta (19–29 Hz) band activities, to identify the clusters (time window and electrode) showing SPE and FPE. To this end, we calculated *t*-values at each time point and each electrode at a particular frequency band (i.e. delta/theta/alpha and low beta/beta). Adjacent points in a cluster were grouped into one or multiple clusters based on a predefined threshold (*P < *.05, cluster level corrected). The summed cluster *t*-value were compared against a permutation distribution, which was generated by randomly reassigning condition membership for each participant (1000 iterations) and computing the maximum cluster mass on each iteration. This approach reliably controls for multiple comparisons at the 2D (time × electrode) cluster level. Similar to the previous study (Haciahmet et al. 2023), we found a positive cluster in the beta band (19–29 Hz) in SP-Group. A negative cluster at the delta/theta band (2–7 Hz) and a negative cluster at the alpha band (8–18 Hz) were also identified for SP-Group. The FP-Group showed only a negative cluster at the alpha and low beta band (8–18 Hz). The time-varying signals of alpha and low beta band (8–18 Hz) clusters were extracted for point-by-point correlation analysis in FP-Group (F7, F3, FZ, FT7, FC3, C3,T3) and SP-Group (FCZ, FC4, C3,CZ, C4,CPZ, CP4), respectively.

### Canonical correlation analysis

We conducted canonical correlation analyses to assess the relationships between interpersonal traits and friend-priority effect shown in behavioral responses. This analysis sought to find the maximal correlation between two matrixes (that is, a linear combination of one set of variables). We used R package (https://cran.r-project.org/web/packages/ccaPP/index.html) *ccaPP* (Alfons et al. 2016) to assess the canonical associations between interpersonal traits/relationships (IRI, self-construal, friendship) and behavioral response of friend associations (i.e. response accuracy and RTs to friend–shape pairs in the matching condition, d’ and β of friend associations). Permutation tests with 1000 iterations were conducted to assess the significance of estimated associations.

## Results

### Classification of FP-Group and SP-Group based on behavioral performances

We examined the difference in response efficiencies related to correct self-shape and friend–shape associations in each participant to identify FP-Group and SP-Group, respectively. We found 216 participants (22% of the whole testing sample) who showed smaller response efficiencies (i.e. better performances) when responding to correct friend–shape than self–shape associations. These participants were thus included in the FP-Group. To characterize and contrast the cognitive and neural processes supporting friend prioritization, we selected the top 22% of the testing sample who showed smaller response efficiencies (i.e. better performances) when responding to correct self–shape than friend–shape associations as controls (i.e. the SP-Group, see Supplementary Table S1 for demographic information of the two groups). We conducted bootstrapped analyses (Davison and Hinkley 1997) of RTs and response accuracies to illustrate the differences in behavioral performances between FP-Group and SP-Group (Fig. 1b). SP-Group responded faster with higher accuracies to correct self–shape than friend–shape associations, replicating previous findings (Sui et al. 2012, 2013, 2023, Golubickis et al. 2017, Liang et al. 2022, Scheller and Sui 2022). In contrast, FP-Group responded faster with higher accuracies to correct friend–shape than self–shape associations. In addition, both SP-Group and FP-Group performed worse when responding to correct stranger-shape than self/friend–shape associations.

To further verify the distinct patterns of behavioral performances in FP-Group and SFP-Group, we conducted mixed-designed ANOVAs of response accuracies and RTs to correct word–shape associations with Label (Self, Friend, vs. Stranger) as a within-subjects variable and Group (SP-Group vs. FP-Group) as a between-subjects variable. The results revealed significant interactions between Label and Group on both response accuracies and RTs [*F*(2860) = 163.00/319.89, *P*’s < .001, *η_p_^2^* = 0.275/0.427, 90% CI = (0.234, 0.313)/]/(0.387, 0.461)]. Post hoc comparisons with Bonferroni corrections further verified faster and more accurate responses to correct self–shape than friend–shape associations [mean difference = −−83.17 ms/12.4%, *P*’s < .001, 95% CI = (−88.60, −77.75)/(11.2%, 13.6%)] and stranger–shape pairs [mean difference = −97.97 ms/18.7%, *P*’s < .001, 95% CI = (−105.83, −90.12)/(16.7%, 20.8%)] in SP-Group. However, FP-Group responded faster and more accurately to correct friend–shape than self–shape associations [mean difference = −19.39 ms/5.6%, *P*’s < .001, 95% CI = (−24.82, −13.96)/(4.4%, 6.8%)] and stranger–shape pairs [mean difference = −53.53 ms/10.2%, *P*’s < .001, 95 CI = (−60.89, −46.16)/(8.2%, 12.2%) Fig. 1c and d)] These behavioral results identified a group of individuals who prioritized friends over the self in the perceptual matching task.

To test whether participants’ prior experiences with the friend might contribute to the differences in behavioral responses to friend–shape than self–shape associations between SP-Group and FP-Group, we asked participants to report how long they had known the friend when being tested. A two-sample *t*-test of the reported durations did not show a significant group difference [mean ± SE = 65.35 ± 2.9 vs. 64.93 ± 2.9 months for SP-Group and FP-Group, respectively, *t* (413) = 0.099, *P* = .921, Cohen’s *d* = 0.010]. This result indicates that friend-prioritization or self-prioritization shown in behavioral responses in the perceptual matching task cannot be simply attributed to the friendship duration.

### Distinct cognitive processes underlying friend- and self-prioritization

Next, we examined latent cognitive processes underlying friend- and self-prioritization in behavioral performances in the shape-label matching task. We first conducted signal detection theory analyses of behavioral responses to assess participants’ perceptual sensitivity to correct versus incorrect word–shape associations [i.e. discriminability (d’)] and propensity to respond to stimuli as correct word–shape associations [i.e. a response bias (β) to make an affirmative judgment] by integrating hit (i.e. *yes* responses to correct word–shape associations) and false alarm (i.e. “yes” responses to incorrect word–shape associations) when responding to word–shape pairs in each condition. ANOVA of d’ and β showed significant interactions between Label and Group [*F* (2860) = 240.71/43.96, *P*’s < .001, *η_p_^2^* = 0.359/0.093, 90%CI = (0.318, 0.396)/(0.063, 0.123), Fig. 1e and f]. Post hoc comparisons with Bonferroni corrections revealed greater d’ in response to self-shape than friend–shape/stranger-shape pairs mean difference = 0.765/0.794, *P*’s < .001, 95% CI = (0.682, 0.848)/(0.693, 0.894)] but no significant difference in d’ between friend–shape and stranger–shape pairs in SP-Group [mean difference = 0.029, 95% CI = (−0.066, 0.124), *P* > .99]. Nevertheless, FP-Group showed greater d’ when responding to friend–shape than self–shape/stranger–shape associations [mean difference = 0.423/0.452, *P*’s < .001, 95% CI = (0.340, 0.506)/(0.357, 0.548)] but no difference in d’ between self-shape and stranger–shape pairs [mean difference = 0.029, 95% CI = (−0.071, 0.130), *P* > .99]. These results yield evidence for the best sensitivity in response to friend–shape pairs in FP-Group but to self–shape pairs in SP-Group.

One sample *t*-tests (two tailed) of β-values showed that β-value was significantly smaller than 1 only for responses to self–shape pairs in SP-Group [*t* (215) =  −20.84, *P* < .001, Cohen’s *d* = −1.418, 95% CI = (−1.606, −1.23)]. In contrast, β-value was significantly smaller than 1 only for responses to friend–shape pairs in FP-Group [*t* (215) = −10.89, *P* < .001, Cohen’s *d* = −0.741, 95% CI = (−0.891, −0.590)]. These results further highlighted an affirmative response bias when responding to friend–shape pairs in FP-Group but to self–shape pairs in SP-Group.

We further conducted HDDM analyses of RTs and response accuracies to elucidate latent cognitive processes contributing to the distinct response patterns observed in FP-Group and SP-Group. This analysis focused on four model parameters, including drift rate (*v*, an index of speed and quality of information acquisition), starting point (*z*, a time point where evidence accumulation begins), boundary (*a*, an index of how much evidence is sampled before a decision is made), and nondecision time (*t*_0_, an index of the duration of all nondecisional processes, such as perceptual process and motor execution) (White and Poldrack 2014). We tested 15 candidate theoretical models based on whether each of the four parameters was influenced by different conditions (see “Methods” section for details) and selected the winning one (Model 1) through model comparison for further analyses (see Supplementary Table S2 for details).

**Figure 2.**
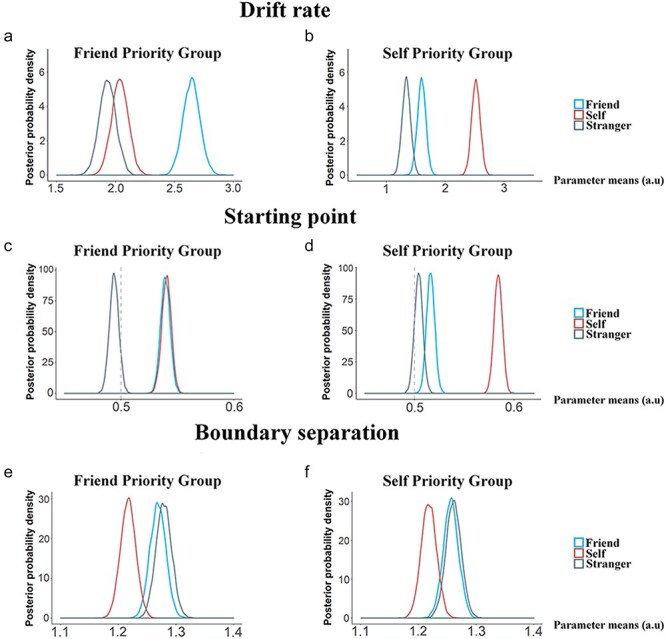
Results of DDM analyses of behavioral performance during the word–shape matching task. (a) and (b) Group-level posterior probability densities for means of drift rate in FP-Group and SP-Group. (c) and (d) Group-level posterior probability densities for means of starting point in FP-Group and SP-Group. (e) and (f) Group-level posterior probability densities for means of boundary separation in FP-Group and SP-Group. The abscissa represents the normalized parameter means estimated by Markov Chain Monte Carlo, which can be arbitrary and have no unit. The ordinate represents the posterior probability densities of a given value.

The results of HDDM analyses identified two opposite patterns of the posterior distribution of drift rates related to self- and friend–shape associations in FP-Group and SP-Group. Specifically, in FP-Group, the posterior distribution of drift rates was greater for friend–shape than self–shape associations [*P*_Bayes_ (self-match < friend-match) = 1] but did not differ between self-shape and stranger–shape associations [*P*_Bayes_ (self-match < stranger-match) = 0.151, Fig. 2a]. This pattern contrasted sharply with that observed in SP-Group, which was characterized with a greater posterior distribution of drift rates for self–shape than friend–shape associations [*P*_Bayes_ (self-match > friend-match) = 1] and for friend-shape compared to stranger–shape associations [*P*_Bayes_ (stranger-match < friend-match) = 0.995, Fig. 2b]. These results implicated faster information acquisition when responding to friend–shape pairs in FP-Group but to self–shape pairs in SP-Group. The results of HDDM analyses also revealed higher starting point in response to correct self–shape than friend–shape associations in SP-Group [*P*_Bayes_ (self-match > friend-match) = 1, Fig. 2d] but not in FP-Group [*P*_Bayes_ (self-match > friend-match) = 0.57, Fig. 2c]. Thus, FP-Group was also characterized by the overlapping time points where evidence accumulation begins during responses to self–shape and friend–shape pairs.

The DDM analyses did not show significant group differences in the other two model parameters. The posterior distribution of boundary separations was lower for correct self–shape than friend–shape associations in both SP-Group and FP-Group [*P*_Bayes_ (self-match < friend-match) = 0.997/0.982, Fig. 2e and f], suggesting that less amount of evidence was required to make judgments on self–shape than friend–shape pairs in both groups. Nondecision time was significantly shorter for match than nonmatch word–shape associations in both SP-Group and FP-Group [*P*_Bayes_ (match < nonmatch) = 1/1], indicating faster nondecisional processes when responding to match than nonmatch associations, consistent with the previous findings (Golubickis et al. 2017).

### Evoked phase-locked brain activities characterizing friend-prioritization and self-prioritization

ERPs in response to the word–shape pairs were characterized by an early negative activity at 100–150 ms (N1) and a positive activity at 180–240 ms (P2) at the frontal/central electrodes followed by a negative response at 240–300 ms (N2).

**Figure 3.**
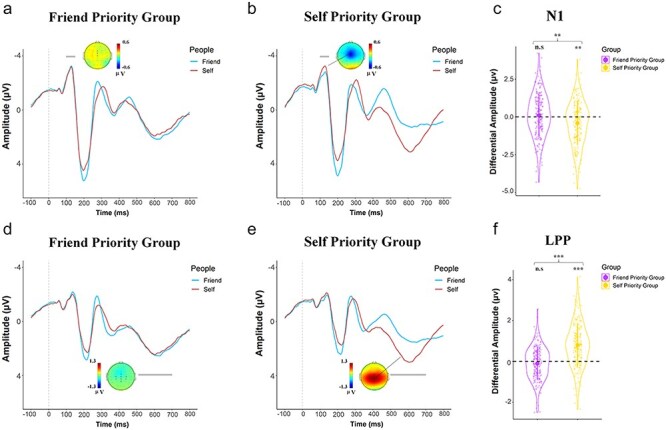
Illustration of ERP results indicating the self-priority effect. (a) and (b). Grand average ERPs at the midline frontalarea in response to the correct label–shape associations in FP-Group and SP-Group, respectively. (c) The differential N1 amplitudes to self–shape (minus friend–shape) stimuli in FP-group and SP-Group , respectively. The voltage topographies show scalp distributions of the maximum differential N1 (correct self–shape associations vs. friend–shape associations). (d) and (e) Grand average ERPs at the central and posterior area in response to the correct label–shape associations in FPGroup and SP-Group, respectively. (f) The differential LPP amplitudes to self-shape (minus friend–shape) stimuli in FP-group and SP-Group , respectively. The voltage topographies show scalp distributions of the maximum differential LPP (correct self–shape associations vs. friend–shape associations). Shown are group means (big dots), standard deviations (bars), measures for each individual (small dots), and distribution (violin shape). ** *P* < .01; *** *P* < .001; n.s = not significant.

**Figure 4.**
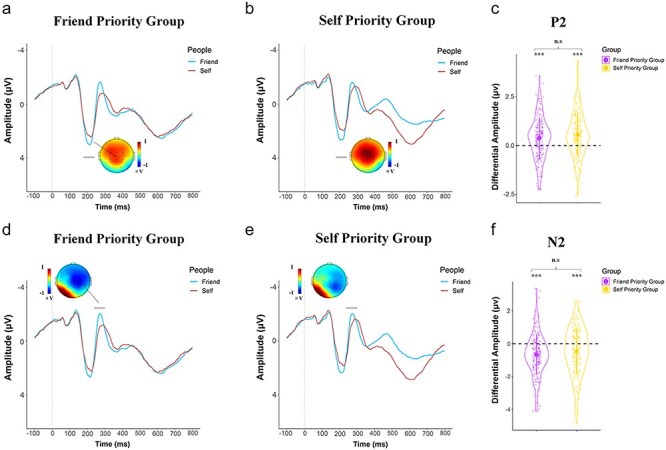
Illustration of ERP results indicating the friend-priority effect. (a) and (b). Grand average ERPs at the frontal to central areas in response to the correct label–shape associations in FP-Group and SP-Group, respectively. (c) The differential P2 amplitudes to friend-shape (minus self-shape) stimuli in FP-group and SP-Group, respectively. The voltage topographies show scalp distributions of the maximum differential P2 (correct friend–shape associations vs. self–shape associations) (d). (e) Grand average ERPs at the right frontal and central area in response to the correct label–shape associations in FP-Group and SP-Group, respectively. (f) The differential N2 amplitudes to friend-shape (minus self-shape) stimuli in FP-group and SP-Group, respectively. The voltage topographies show scalp distributions of the maximum differential N2 (correct friend–shape associations vs. self–shape associations) (g). (h) Grand average ERPs the left posterior and occipital areas in response to the correct label–shape associations in FP-Group and SP-Group, respectively. (i) The differential P3 amplitudes to friend–shape (minus self–shape) stimuli in FP-group and SP-Group, respectively. The voltage topographies show scalp distributions of the maximum differential P3 (correct friend–shape associations vs. self–shape associations) over the left posterior and occipital regions. Shown are group means (big dots), standard deviations (bars), measures for each individual (small dots), and distribution (violin shape). ** *P* < .01; *** *P* < .001; n.s = not significant.

There were also two positive activities at 320–360 ms (P3) over the parietal region and at 500–700 ms (LPP) over the central–parietal regions (Figs 3 and 4). Based on our behavioral results, our EEG data analyses focused on neural responses to correct self–shape and friend–shape associations.

We first conducted ANOVAs of the mean ERP amplitude with Label (Self vs. Friend) as a within-subjects variable and Group (SP-Group vs. FP-Group) as a between-subjects variable. ANOVAs of the N1 and LPP amplitudes showed significant interactions of Label × Group [*F*(1416) = 8.40/116.34, *P* = .004 and <.001, η_p_^2^ = 0.020/0.219, 90% CI = (0.004, 0.047)/(0.163, 0.273)]. Post hoc comparisons with Bonfereroni corrections revealed that, self-shape compared to friend–shape pairs elicited larger N1 (more negative) and LPP (more positive) amplitudes in SP-Group [(mean difference = −0.325/1.101, *P* = .017/<.001, 95% CI = (−0.612, −0.039)/(0.887, 1.316), Fig. 3b and e]. However, the N1 and LPP amplitudes did not differ significantly between self–shape and friend–shape pairs in FP-Group [mean difference = 0.120/−0.137, *P* > .99/= 0.559, 95% CI = (−0.169, 0.409)/(−0.354,0.079), Fig. 3a and d]. ANOVAs of LPP peak latencies also showed a significant interaction of Label × Group [*F*(1416) = 112.66, *P* < .001, η_p_^2^ = 0.213, 90% CI = (0.158, 0.268)], as the LPP peaked earlier to self-shape than friend–shape pairs in SP-Group [656 vs. 693 ms, mean difference = −36.94, *P* < .001, 95% CI = (−43.31, −30.58)] but not in FP-Group [669 vs. 670 ms, mean difference = −0.74, *P* > .99, 95% CI = (−7.16, 5.69)].

ANOVAs of the P2, N2, and P3 amplitudes showed significant main effects of Label [*F* (1416) = 84.84/71.27/143.08, *P*’s < .001, η_p_^2^ = 0.169/0.146/0.256, 90% CI = (0.118, 0.222)/(0.098, 0.198)/(0.198, 0.311)]. Friend-shape compared to self–shape pairs elicited greater P2, N2, and P3 amplitude in both FP-Group and SP-Group [mean difference = 0.717/−0.553/0.847, *P*’s < .001, 95% CI = (0.564, 0.870)/(−0.682,-0.424)/(0.707,0.986), see Fig. 4]. Furthermore, there was a significant interaction of Label × Group on the P3 amplitude [*F*(1416) = 7.96, *P* = .005, η_p_^2^ = 0.019, 90% CI = (0.003, 0.046)], suggesting that the enlarged P3 amplitudes to friend–shape compared to self–shape pairs were more salient in FP-Group than in SP-Group.

Previous ERP findings showed posterior N1 (147–167 ms) showed greater amplitude in response to self–shape pairs compared to friend–shape pairs (Sui et al. 2023). We replicated that the amplitude of the posterior N1 was greater in response to self–shape (vs. friend–shape) associations at the left occipitotemporal electrodes (Tp7 and T5) in both SP-Group and FP-Group [*F*(1416) = 20.06, *P* < .0001, η_p_  ^2^ = 0.046, 90% CI = (0.019 0.08), FP-Group/SP-Group mean difference = −0.222/−0.290, *P* = .04/.002, 95% CI = (−0.437, −0.006)/(−0.504, −0.077)]. Furthermore, similar to the previous study, we analyzed the trial-by-trial correlations between neural activities and DDM parameters with a model that regressed the N1 onto drift rate and the P3 onto boundary separation was estimated (Sui et al. 2023). Trial-by-trial correlation analyses discovered strong evidence for negative correlations between the N1 amplitude and drift rate [*P*’s_Bayes_ (drift rate ∼ N1 < 0) > 0.99/=0.928/ > 0.99/ > 0.99] and between the P3 amplitude and boundary separation and (*P*’s_Bayes_ [boundary separation ∼ P3 < 0) > .99] in response to both self–shape and friend–shape associations in both SP-Group and FP-Group. These results are consistent with the previous findings (Sui et al. 2023) and suggest distinct functional roles of the N1 and P3 activities in facilitation of information accumulation and evidential requirement during decision-making related to self–shape associations, which, however, were common for SP-Group and FP-Group.

**Figure 5.**
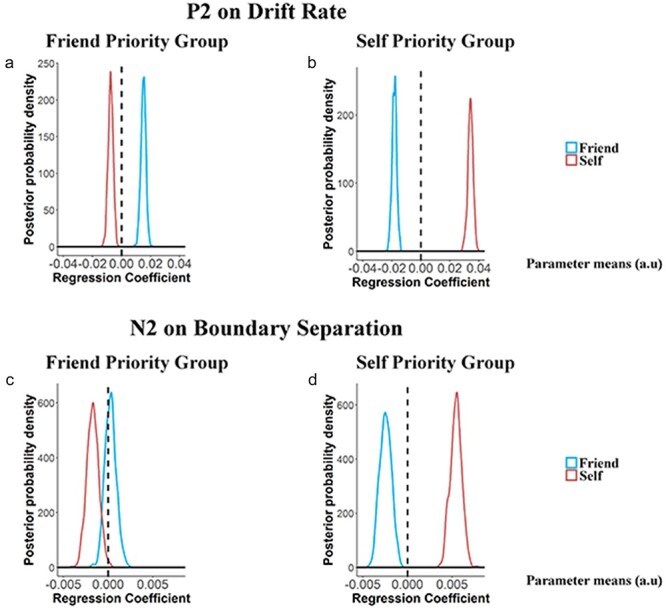
(a and b) Group-level posterior probability densities for means of regression coefficient distributions of the P2 on drift rate in FP-group and SP-group, respectively. (c and d) Group-level posterior probability densities for means of regression coefficient distributions of the N2 on boundary separation in response to self- and friend-shape associations in FP-Group and SP-Group, respectively.

Interestingly, the trial-by-trail correlation analyses in SP-Group showed positive correlations between the P2 amplitudes and drift rate and between the N2 amplitudes and boundary separation [*P*_Bayes_ (drift rate ∼ P2 > 0) > .99/*P*_Bayes_ (boundary separation ∼ N2 > 0) > .99] in response to self–shape associations but negative correlations [*P*_Bayes_ (drift rate ∼ P2 < 0) > .99/*P*_Bayes_ (boundary separation ∼ N2 < 0) > .99, Fig. 5] in response to friend–shape associations. By contrast, FP-group showed evidence for positive correlations between the P2 amplitudes and drift rate and between the N2 amplitudes and boundary separation when responding to friend–shape associations [*P*_Bayes_ (drift rate ∼ P2 < 0) > .99)/*P*_Bayes_ (boundary separation a ∼ N2 > 0) = .652, Fig. 5] but negative correlations when responding to self–shape associations [*P*_Bayes_ (drift rate ∼ P2 < 0) > .99/*P*_Bayes_ (boundary separation ∼ N2 < 0) > .99]. These results suggest similar couplings between the P2/N2 activities and the cognitive modules (i.e. drift rate/ boundary separation) supporting the prioritized label–shape associations in both SP-Group and FP-Group, that is, the greater P2 amplitude was associated with faster information acquisition whereas the increased N2 amplitude was linked to less amount of required evidences for decision making when responding to associations of the prioritized target with a shape (i.e. the self–shape association for SP-Group, and the friend–shape association for SP-Group).

### Friend-prioritization-specific modulation of induced nonphased locked brain activities

**Figure 6.**
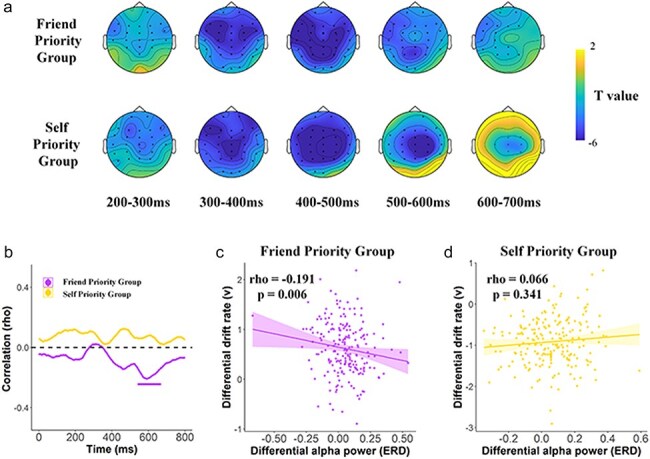
The results of time-frequency analyses. (a) Topographies of the differential time-frequency power between correct self–shape vs. friend–shape associations in alpha band (8–18 Hz) in FP-Group and SP-Group. (b) Point-by-point correlations between differential alpha power (correct self–shape vs. friend–shape associations) and corresponding differential drift rates in FP-Group and SP-Group. (c) and (d) Correlations between differential alpha power around 542–668 ms and information acquisition bias (drift rate) in FPGroup (c) and SP-Group (d).

We further estimated differences in nonphase-locked neural responses to correct self–shape and friend–shape associations by conducting time-frequency analyses of EEG signals. The cluster-based permutation test between self–shape and friend–shape associations in SP-Group revealed a negative cluster at 290–800 ms at the frontal/central electrodes (cluster *P* = .002, Bonferroni corrected *P* = .006) in the delta/theta activities (2–7 Hz) and a positive cluster at 529–687 ms at the frontal/central electrodes (cluster *P* = .042, Bonferroni corrected *P* = .126) in the high beta activities (19–29 Hz), similar to previous findings (Haciahmet et al. 2023). However, the same analyses of delta/theta/beta activities failed to discover any significant cluster between self–shape and friend–shape associations in FP-Group. The analyses of alpha and low beta activities (8–18 Hz) showed that the contrast of self–shape vs. friend–shape associations revealed a significant negative cluster at 212–800 ms at the left frontal and central electrodes in FP-Group (cluster *P* = .002, Bonferroni corrected *P* = .006, Fig. 6a) and at 85–671 ms at the frontal/central electrodes in SP-Group (cluster *P* = .002, Bonferroni corrected *P* = .006), suggesting similar modulations of the alpha/low beta oscillations by self–shape versus friend–shape association in FP-Group and SF-Group.

We further examined relationships between alpha/low beta oscillations and latent cognitive mechanisms (as indexed by the drift rate) underlying friend-/self-prioritization as indexed by the HDDM parameters. The point-by-point correlation analyses discovered a significant negative cluster in FP-Group (*P*’s < .05 within successive 100 time points, uncorrected, Fig. 6b), but no significant cluster was found in SP-Group. The distinct brain-cognition coupling patterns between FP-Group and SP-Group were further confirmed by a modulation analysis [*F*(1, 414) = 6.28, *P* = .013, η_p_^2^ = 0.015, 90% CI = (0.002, 0.040)]. In FP-group, greater decrease of alpha/low beta power in response to friend–shape (vs. self–shape) associations at 542–668 ms predicted faster evidence accumulation (as indexed by larger drift rate) when responding to friend–shape (vs. self–shape) associations (rho = −0.191, *P* = .006, Fig. 6c), whereas such nonsignificant correlation was identified in SP-Group (rho = 0.066, *P* = .341, Fig. 6d).These results suggest a friend-prioritization-specific modulation of alpha/low beta oscillations in FP-Group.

### Relationships between friend-prioritization and interpersonal traits

**Figure 7.**
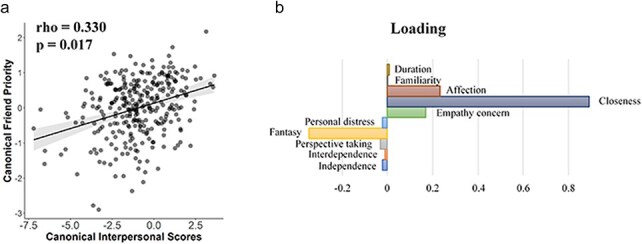
Results of correlation analyses of canonical traits and performances in the perceptual matching task. (a) Canonical correlation between interpersonal trait measurements (IRI, Self-construal, Friendship assessment) and behavioral performances in the perceptual matching task (ACC, RT, d’ and β). (b) The standardized canonical coefficients (factor loading) of each dimension among subjective measurement.

To test whether friend-prioritization in behavioral performances in the perceptual matching task was associated with interpersonal traits across individuals, we calculated a canonical interpersonal trait score by combining IRI scores (empathy concern, perspective taking, fantasy, personal distress), self-construal scores (interdependent and interdependent self construals), friendship indices (time, closeness, likability, and familiarity related to the friend). Canonical friend-priority was assessed by combining behavioral performances in the perceptual matching task (response accuracies, RTs, sensitivity, and response bias). In the canonical friend-priority matrix, the factor loadings of response accuracies, RTs, sensitivity, and response bias were 0.742, −0.662, −0.04, and −0.1, respectively, suggesting that larger canonical scores indicate better performance (i.e. higher accuracy and faster reaction time) in response to correct friend–shape associations. The predictive loading of canonical interpersonal scores results suggested that higher empathy concern, more likability and closer with the friend predicted larger friend-priority effect in the perceptual matching task (See Fig. 7b). Importantly, a canonical correlation analysis revealed a significant association between canonical interpersonal trait scores and canonical friend priority across all participants (a permutation test, rho = 0.330, *P* = .017, Fig. 7a), suggesting larger friend-priority in behavioral performances in individuals with greater interpersonal trait scores.

### Relationships between friend-prioritization and social trust/cooperative tendency

**Figure 8.**
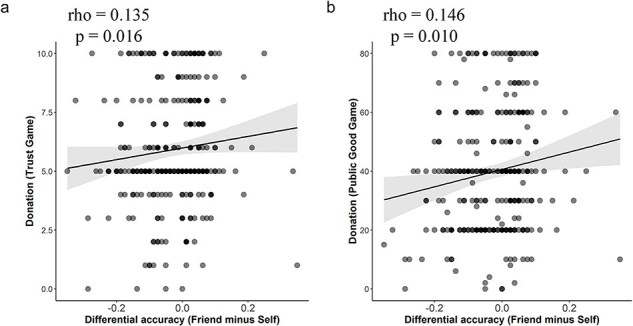
Results of correlation analyses of performances in the perceptual matching task and the economic games. (a) Correlations between cooperative behaviors in the trust game and the differential response accuracy between friend–shape vs. self–shape associations. (b) Correlations between cooperative behaviors in the Public goods game and the differential response accuracy between friend–shape vs. self–shape associations.

Finally, we tested whether friend-prioritization in the perceptual matching task was able to predict individuals’ social trust and cooperative tendencies, which were estimated using the trust game (Berg et al. 1995) and public goods game (Ledyard 1995), respectively. We first compared group-level differences in social trust and cooperative tendencies by conducting a mix-design repeated-measurement ANOVA of monetary units with Game (Trust game vs. Public good game) as a within-subjects factor and Group (SP-Group vs. FP-Group) as a between-subjects factor. The results revealed only a significant main effect of Group [*F* (1312) = 5.087, *P* = .025, η2 = 0.016, 90% CI = (0.001,0.411)] due to more contributions in the games in FP- Group than in SP-Group. We then examined individual-level covariations of performances in the perceptual matching task and social trust/tendency differences. The results of correlation analyses showed that larger response accuracies to friend–shape (vs. self–shape) pairs predicted more monetary units contributed in the trust game and public goods game across all participants (rho = 0.135/0.146, *P* = .016/0.010, 95% CI = (0.032, 0.237)/(0.046, 0.249), Fig. 8]. These results suggest possible associations between friend-prioritization in behavioral responses in the perceptual matching task and social trust and cooperative tendencies.

## Discussion

The present study investigated individual differences in prioritization of self-related information by examining whether information about friends may be prioritized in a perceptual matching task in subpopulations. We recorded behavioral and EEG data in a perceptual matching task from a large sample and identified a subpopulation whose behavioral responses in the perceptual matching task were characterized by friend-prioritization. Furthermore, our EEG/ERP results revealed specific and nonspecific neural correlates of cognitive processes underlying friend-prioritization and self-prioritization. These findings brought several contributions to our understanding of perceptual processing of social information about the self and close others in adults.

First, while previous studies repeatedly reported behavioral evidence that people prioritize self-related stimuli compared with those related to others in different tasks (Symons and Johnson 1997, Conway and Pleydell-Pearce 2000, Brédart et al. 2006, Cunningham et al. 2008, Turk et al. 2011, Sui et al. 2012), the current study showed behavioral evidence that, in the label-shape matching task, about one-fifth of a Chinese university student population showed advantages in behavioral response efficiencies pertaining to correctly learned friend–shape compared to self–shape associations. Friend-prioritization in behavioral performances was verified in a sample which was larger than the samples in previous studies that tested self-prioritization using the same task (e.g. Sui et al. 2012, Golubickis et al. 2017, Macrae et al. 2018, Svensson et al. 2022). Interestingly, the proportion of the individuals who showed friend-prioritization (22%) is close to the percentage of altruistic individuals (Fehr and Schmidt 1999) whose behaviors in economic games were not compatible with the self-interested model (Gintis et al. 2003). Our behavioral findings provide group-level evidence that friend-prioritization is not a causal case and characterizes perceptual processing in a subpopulation who prioritize friends over oneself in a perceptual matching task. Moreover, we showed that individuals who showed friend-prioritization in the perceptual matching task also exhibited enhanced social trust and cooperative tendencies relative to those showing self-prioritization. These findings suggest consistent other-oriented propensities at multiple-levels of cognitive processes in this subpopulation.

Second, the analyses of the behavioral performances in the shape-label matching task uncovered distinct latent cognitive processes involved in friend-prioritization and self-prioritization, respectively. The results of signal detection theory analyses of behavioral responses revealed the best sensitivity and an affirmative response bias in response to learned friend–shape (vs. self–shape or stranger–shape) associations in FP-Group. SP-Group, however, exhibited the best sensitivity and an affirmative response bias in response to self-shape (vs. friend–shape or stranger–shape) associations. Consistent with these results, the results of HDDM analyses provided further evidence for faster information acquisition when learning friend-related associations as indexed by larger drift rates when responding to friend–shape associations in FP-Group. In contrast, SP-Group showed faster information acquisition when learning self–shape associations. Faster information acquisition for self–shape than stranger pairs was reported in a previous work (Golubickis et al. 2017, Svensson et al. 2022, Sui et al. 2023). Another study found a similar trend of faster information acquisition when responding to self-owned relative to friend-owned items in an object-categorization task (Golubickis et al. 2018). While the priori response bias to self–shape over friend–shape associations in SP-Group is consistent with the previous findings, FP-Group in our work did not show a significant response bias for friend–shape associations. Therefore, our HDDM results dissociated two distinct latent mechanisms underlying the friend-prioritization effect, i.e. facilitated acquisition of friend-related information and unbiased initial response to self-related stimuli. These findings provide potential cognitive bases for assessment of individuals’ social orientations in a continuous dimension from egocentrism to altruism (Haslam et al. 2020).

Third, our ERP results revealed FP-Group/SP-Group specific and nonspecific modulations of neural responses to self/friend–shape associations. The amplitudes of the anterior N1 at the frontal/central electrodes and the LPP at the central/parietal electrodes were larger in response to correct self–shape (vs. friend–shape) associations. The enhanced N1/LPP responses to self-related stimuli were verified in SP-Group and are consistent with the previous findings (Sui et al. 2023). However, FP-Group showed comparable N1/LPP amplitudes in response to self–shape and friend–shape associations, indicating comparable neural processes in both an early and a late time windows. The frontal N1 is supposed to reflect the effect of voluntarily selective attention toward task-relevant stimuli (Näätänen 1982) and is sensitive to motivation-related processing of stimuli (Gable and Harmon-jones 2011). The LPP is involved in elaborative coding of stimulus significance (Weinberg and Hajcak, 2011, Hajcak and Foti 2020) and showed increased amplitudes in response to self-related (vs. other-related) stimuli such as name and face (Sui et al. 2009, Tacikowski and Nowicka 2010, Fan and Han 2018). It has been assumed that the enlarged N1 response to self–shape association compared to friend- or stranger–shape associations in the label-shape matching task benefits the initial perception of the label of the self and its integration with a shape due to the augmented attentional processing elicited by personally meaningful stimuli during the early stages of decision-making (Sui and Humphreys 2015, Humphreys and Sui 2016). If this analysis is correct, our findings indicate that the early attentional process may bias the labels of friend/self and their integration with shapes similarly in FP-Group. In addition, FP-Group may employ similar cognitive effort during late elaborative coding of label–shape associations when responding to friend-related and self-related stimuli.

Our ERP results also discovered that the amplitudes of mid-latency neural responses including the P2 and N2 components were enlarged in response to friend–shape than self–shape associations and these effects were observed in both FP-Group and SP-Group. The results are consistent with the previous findings that friend faces (vs. self faces) elicit larger P2 and N2 amplitudes (Keyes et al. 2010). The P2 amplitude is modulated by social distance and fairness consideration of friends (Wu et al. 2011) and outcome valence (i.e. win or loss) of friends (Kwak et al. 2020). The anterior N2 component is sensitive to the strength of conflicting action imperatives and subserves the cognitive control function (e.g. strategic monitoring and control of motor responses (Azizian et al. 2006, Folstein and Van Petten 2008). Our results suggest similar enhanced neural encoding of friend-related (vs. self-related) stimuli and neural processes underlying cognitive control mediated by the mid-latency neural responses in FP-Group and SP-Group, which, however, do not necessarily lead to friend-prioritization in behavioral responses.

Finally, our trial-by-trial regression analyses of EEG data revealed specific and nonspecific neural correlates of cognitive processes (e.g. information acquisition speed) underlying friend-prioritization and self-prioritization. Our analyses of nonphased locked neural activities found decreased alpha and low beta responses in response to self–shape vs. friend–shape associations in both FP-Group and SP-Group. While previous EEG research found that higher degree of neural integration (i.e. higher power-law exponent and longer autocorrelation window) during resting-state EEG were related to a stronger increase in the self-prioritization effect (Kolvoort et al. 2020), our results suggested that the modulations of stimulus evoked neural oscillations may also contribute to the self-prioritization effect. Because recent research has shown evidence that a lower intrinsic prestimulus alpha power yields higher degrees of neural excitability (Kolvoort et al. 2020, Iemi et al. 2022, Northoff et al. 2024), we estimated if the modulations of alpha and low beta powers by responses to self–shape vs. friend–shape associations were produced by the intrinsic pre-stimulus alpha power. We tested whether the difference in the intrinsic prestimulus alpha power was associated with the decreased alpha and low beta power in response to self–shape vs. friend–shape associations but did not find any significant results in either FP-Group or SP-Group (see Supplementary Fig. S1). Thus, there was no evidence that the decreased alpha and low beta power in response to self–shape vs. friend–shape associations observed in our work might be generated by the intrinsic prestimulus alpha responses. The decreased beta responses were consistent with previous findings of desynchronized beta power in the contrast of correct self–shape versus stranger–shape associations in the label-shape matching task (Haciahmet et al. 2023), suggesting a self-specific modulation of beta oscillations. Decreased alpha power reflects a general attention demand and active cognitive control, whereas increased alpha power is thought to reflect inhibitory of these cognitive functions (Klimesch et al. 2007). Our findings support the idea that self-related information increased internally directed attention but inhibition of external attention than other-related information (Mu and Han 2010). However, only the decrease of alpha/low beta power in response to friend–shape (vs. self–shape) associations in FP-Group predicted larger drift rates as an index of faster evidence accumulation when responding to friend–shape (vs. self–shape) associations. It is likely that enhanced attention and cognitive control were specifically engaged in FP-Group to speed information accumulation from friend-related stimuli. This effect, though occurred after 500 ms after stimulus onset, may facilitate motor response selection and execution when responding to friend–shape associations.

Our trial-by-trial regression analyses of EEG data also found neural correlates of speed of information acquisition that were specific to prioritized target. The greater P2/N2 amplitudes were associated with faster information acquisition and less amount of required evidences for decision making when responding to self–shape associations in SP-Group but when responding to friend–shape associations in FP-Group. These results implicate that the neural processes in the P2/N2 time windows may support information acquisition and modulation of evidence accumulation from prioritized targets in each group regardless that the prioritized target was different in FP-Group and SP-Group. In addition, our trial-by-trial regression analyses of EEG data showed similar coupling between the N1/LPP amplitudes and with drift rate/boundary separation related to both self-shape and friend–shape associations in both SP-Group and FP-Group. If these results reflect facilitation of information acquisition from label–shape associations by voluntary attention and increased accessibility of shape–label relations in working memory (Sui et al. 2023), these functional associations may not be target specific. In other words, similar phase-locked neural activities in the early and late time windows in the processing stream may be engaged to support correct judgments of shape associations with prioritized targets (either self in SP-Group or friend in FP-Group).

It should be noted that friend-prioritization in behavioral performances in the perceptual matching task varied across individuals. We found that a canonical interpersonal score integrating higher empathy concern, more likability, and closer with the friend predicted stronger friend-prioritization in behavioral performances pertaining to correct friend–shape associations in the label–shape matching task. This result implicates personal traits and interpersonal relationships as a potential social drive of the advantage of perceptual processing of information and relevant decision-making related to best friends over the self. Specifically, traits such as empathy concern might enhance individual’s ability to process friend-related information and to respond to friends’ emotional needs quickly. Such social information processing and behavioral in turn may reinforce the importance of close social bounds and intrinsic satisfaction derived from social interactions. Furthermore, we found that friend-prioritization in behavioral responses in the perceptual matching task positively predicted individuals’ social trust and cooperative tendencies estimated using performances in the trust game (Berg et al. 1995) and public good game (Ledyard 1995). This finding suggests a close link between perceptual sensitivity to close friends’ information and own social traits/behaviors in real-life social interactions. Perceptual sensitivity to close friends’ information may also help to foster deep friendships and afford effective social communication.

Taken together, our study identified a subpopulation who showed friend-prioritization in behavioral responses in a perceptual matching task. Our analyses of both behavioral and EEG data further uncovered the cognitive and neural underpinnings of friend-prioritization, contrasting those supporting self-prioritization in behavioral responses in the perceptual matching task. Future research should extend the current work by examining whether friend-prioritization may be observed in the perceptual processing of stimuli in other domains such as name and face. It is also important to address whether friend-prioritization shown in response to different types of stimuli (e.g. highly familiar or newly learned stimuli) is mediated by similar neural mechanisms. In addition, other neuroimaging techniques such as functional magnetic resonance imaging with high spatial resolution can be used to clarify whether distinct neural circuits are involved in friend-prioritization and self-prioritization during the perceptual learning task. Finally, given the previous findings of cultural differences in cognitive and neural processes of information related to the self and close others (Han and Northoff 2008, Han et al. 2013, Han and Ma 2015, Luo et al. 2024), future research should investigate whether the friend-prioritization effect exists at the group level in other cultural samples and how cognitive and neural underpinnings of friend-prioritization of perceptual processing of social information are shaped by individuals’ cultural experiences.

## Supplementary Material

nsaf009_Supp

## Data Availability

All data and research materials are available at OSF (https://osf.io/c98d4/).
