# Supplementary material for: Cognitive and neural underpinnings of friend-prioritization in a perceptual matching task
Source: Soc Cogn Affect Neurosci. 2025 Jan 20;20(1):nsaf009. doi: 10.1093/scan/nsaf009 (PMC11792655; doi:10.1093/scan/nsaf009)
Supplement: nsaf009_Supp [file nsaf009_supp.zip › scan-24-302-File002.docx]

**Cognitive and neural underpinnings of friend-prioritization in a perceptual matching task**

**Supplementary Information includes:**

**Supplementary Table S1~S2 & Supplementary Figure S1**

**Table S1** Sample sizes for comparisons of different data sets.

|  | FP- Group | SP-Group |
| --- | --- | --- |
| Behavior | 216 (189 females, 20.55 ± 0.87 yrs) | 216 (178 females, 20.44 ± 1.00 yrs) |
| ERPs | 207 (177 females, 20.54 ± 0.87 yrs) | 211 (166 females, 20.44 ± 0.97 yrs) |
| Questionnaire | 210 (163 females, 20.20 ± 0.78 yrs) | 207 (161 females, 20.20 ± 0.76 yrs) |
| Economics Game | 162 (138 females, 20.60 ± 0.89 yrs) | 152 (121 females, 20.50 ± 1.03 yrs) |

Note: Gender and age (mean ± s.d.) are provided.

Table S2. DIC values for model comparisons

| Model label | Hypothesized condition effects on parameters | DIC |
| --- | --- | --- |
| Model 1 | *a,v,z,t_0_* | -93438.83 |
| Model 2 | *a,v,t_0_* | -91349.37 |
| Model 3 | *a,v,z* | -85015.86 |
| Model 4 | *a,z,t_0_* | 59041.61 |
| Model 5 | *v,z,t_0_* | -92963.07 |
| Model 6 | *v,z* | -85855.99 |
| Model 7 | *v,t_0_* | -91284.29 |
| Model 8 | *z,t_0_* | 60962.18 |
| Model 9 | *a,t_0_* | 61303.40 |
| Model 10 | *a,v* | -82827.17 |
| Model 11 | *a,z* | 71378.99 |
| Model 12 | *v* | -84103.18 |
| Model 13 | *z* | 72010.16 |
| Model 14 | *a* | 74191.75 |
| Model 15 | *t_0_* | 49491.23 |


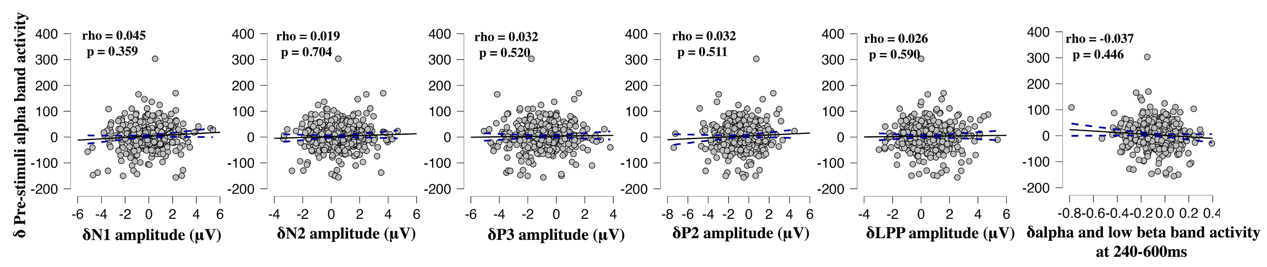


**Figure S1**. Results of the correlation analyses between the differenct in the pre-stimuli alpha band activities (self-shape matching pairs minus friend-shape matching pairs) and the corresponding differential amplitides of the N1, N2, P3, P2, LPP and the alpha and low beta band activity at 240-600ms. There was not any significant result.
